# Effect of propolis on Th2 and Th17 cells: interplay with EtxB- and LPS-treated dendritic cells

**DOI:** 10.1590/1414-431X2023e12659

**Published:** 2023-04-14

**Authors:** B.J. Conti, K.B. Santiago, E.O. Cardoso, F.L. Conte, M.A. Golim, M.T. Cruz, J.M. Sforcin

**Affiliations:** 1 Universidade Estadual Paulista Departamento de Ciências Químicas e Biológicas Instituto de Biociências Botucatu SP Brasil Departamento de Ciências Químicas e Biológicas, Instituto de Biociências, Universidade Estadual Paulista, Botucatu, SP, Brasil; 2 Universidade Estadual Paulista Hemocentro de Botucatu Faculdade de Medicina Botucatu SP Brasil Hemocentro de Botucatu, Faculdade de Medicina, Universidade Estadual Paulista, Botucatu, SP, Brasil; 3 University of Coimbra Faculty of Pharmacy Center for Neurosciences and Cellular Biology Coimbra Portugal Faculty of Pharmacy, Center for Neurosciences and Cellular Biology, University of Coimbra, Coimbra, Portugal

**Keywords:** Dendritic cells, CD4+ T cells, Propolis, Immunomodulation

## Abstract

Dendritic cells (DCs) are antigen-presenting cells that drive the differentiation of T CD4^+^ cells into different profiles according to the nature of the antigen or immunomodulator. Propolis is a resinous product made by bees that has numerous pharmacological properties, including an immunomodulatory action. To assess whether propolis can modulate the activation of CD4^+^ T cells by stimulating DCs with heat-labile enterotoxin B subunit (EtxB) or lipopolysaccharide (LPS), we aimed to elucidate the mechanisms affected by propolis in the differential activation of T lymphocytes. Cell viability, lymphocyte proliferation, gene expression (*GATA-3* and *RORc*), and cytokine production (interleukin (IL)-4 and IL-17A) were analyzed. Propolis, EtxB, and LPS induced a higher lymphoproliferation compared with the control. Propolis induced *GATA-3* expression and, in combination with EtxB, maintained the baseline levels. Propolis alone or in combination with LPS inhibited *RORc* expression. EtxB alone and in combination with propolis increased IL-4 production. Propolis in combination with LPS prevented LPS-induced IL-17A production. These results opened perspectives for the study of biological events that may be favored by propolis by promoting Th2 activation or helping in the treatment of inflammatory conditions mediated by Th17 cells.

## Introduction

Dendritic cells (DCs) exert a crucial role as antigen-presenting cells and in activating adaptive immunity by coordinating effective immune and inflammatory responses (1). Due to constant exposure to a wide range of potentially infectious agents and considering the specific nature of each antigen, DCs contribute to the induction of a polarizing microenvironment appropriate to the pathogen, as revealed by distinct patterns of CD4^+^ T cell phenotypes (2). T cell activation occurs when DCs have been previously activated by antigens, leading to their maturation, which is characterized by increased expression of costimulatory molecules and changes in the chemokine receptors enabling DCs to migrate to organ-draining lymph nodes (3).

Mature DCs express MHC-II molecules that present antigen-derived peptides. The MHC-II-antigen complexes bind to T cell receptors (TCR) on naive CD4^+^ T cells, which can be activated by cytokines in the environment and differentiate into T effector cells: Th1, Th2, Th17, and T regulatory (Treg) cells. Such subsets are characterized by the main transcription factors and cytokines: Th1 cells are associated with T-box transcription factor expressed in T cells (T-bet) and interferon (IFN)-γ production; Th2 cells with GATA-3 and interleukin (IL)-4; Th17 cells with RORγt and IL-17; and Tregs with FoxP3 and TGF-β1. In general, Th1 cells play an important role in fighting intracellular microbial and viral infections; Th2 cells are important in the defense against helminths and allergic diseases; Th17 cells are essential in protecting against bacterial and fungal infections and also in the development of autoimmune diseases; and Tregs play a critical role in controlling the immune response (4-6).

Different immunomodulators are able to affect innate immune cells and T cell activation, proliferation, and migration to inflamed tissues (7). Heat-labile enterotoxin B subunit (EtxB) produced by *Escherichia coli* is a strong mucosal adjuvant that affects DCs maturation and activation, increases the presentation of antigens via MHC class II, and activates a selective lymphocyte differentiation into a Th2 profile (8-10). On the other hand, lipopolysaccharide (LPS), a major component of the outer cell wall of Gram-negative bacteria, is a potent activator of Th17 responses due to its action on DCs (11-13).

Propolis is a resinous product produced by honeybees from wax, saliva, and exudate of buds and barks of plants. It has several pharmacological properties and the possibility of application in the pharmaceutical and food industries, although it has been used in folk medicine since ancient times (14). The immunomodulatory action of propolis on macrophages, monocytes, natural killer cells, DCs, and neutrophils has been widely reported, but its effects on T lymphocytes have been less investigated (15-17).

Research on the mechanisms involved in T cell differentiation and function is crucial for developing new strategies to modulate the immune response and prevent inflammatory diseases (6). Using EtxB and LPS, we investigated the modulatory effects of propolis on human DCs and the mechanisms driving the differentiation of CD4^+^ T cells into a Th2 or Th17 profile by assessing lymphocyte proliferation, transcription factor expression (*GATA-3* and *RORc*), and cytokine production (IL-4 and IL-17A).

## Material and Methods

### Propolis, EtxB, and LPS

Propolis was collected in the Beekeeping Sector of the São Paulo State University (UNESP), Botucatu Campus, and stored at -20°C. Its chemical composition was characterized by gas chromatography-mass spectrometry (GC-MS) and thin-layer chromatography (TLC) (18). Ethanolic extracts of propolis were prepared and diluted in RPMI 1640 culture medium (Cultilab, Brazil) supplemented with 10% fetal bovine serum (FBS) to obtain 5 µg/mL.

EtxB and LPS were purchased from Sigma-Aldrich (USA). The concentrations of propolis, EtxB (2 µg/mL), and LPS (1 µg/mL, isolated from *Escherichia coli* O26:B6) were diluted in RPMI and used in all protocols.

EtxB was first diluted in ultrapure water and propolis was diluted in 70% ethanol. The highest concentration of 70% ethanol (0.013%) used in the assays was equivalent to the highest concentration of propolis.

### Preparation of immune cells

Peripheral blood mononuclear cells (PBMCs) were purified using Ficoll-Paque Plus (GE Healthcare Bio-Sciences, Sweden) from 5 healthy donors (both genders, non-smokers, aged 20-40 years, not sick or using any type of medication). Monocytes (CD14^+^) and naive CD4^+^ T cells were selected using the negative magnetic technique “MACS” (magnetic activated cell sorting; Miltenyi Biotec Inc., USA). Cell viability, as determined by 0.2% trypan blue dye exclusion, was >95% in all experiments.

Monocytes were used for DCs differentiation, and lymphocytes were cryopreserved in RPMI containing 10% FBS and 10% dimethylsulfoxide (DMSO; Sigma-Aldrich) and stored in liquid nitrogen.

This study was approved by the Ethics Committee of Botucatu Medical School (CAAE: 42600915.0.0000.5411) and an informed consent was signed by the blood donors.

### CD14^+^ and CD4^+^ T cells phenotyping

CD14^+^ and CD4^+^ cells were transferred to cytometry tubes (BD Becton Dickinson and Co., USA) and centrifuged at 650 *g* for 10 min at room temperature. The supernatant was discarded and cells were incubated with anti-CD14 monoclonal antibodies (mAbs) conjugated with PerCP/Cy5.5 (0.3 μL) and anti-CD4 conjugated with PerCP/Cy5.5 (0.3 μL) for 30 min (Biolegend, USA). A control tube (autofluorescence) with no labeled cells and an isotypic control tube were included in each test. After incubation, cells were analyzed in a FACS CaliburTM (BD Becton Dickinson and Co.) flow cytometer acquiring a total of 50,000 events.

### DCs generation and phenotyping

DCs were generated from human monocytes after PBMC isolation. Purified monocytes (1×10^6^ cells/mL) were resuspended in complete RPMI 1640 medium plus human recombinant IL-4 (80 ng/mL) and GM-CSF (80 ng/mL) (R&D Systems, USA) for seven days at 37°C and 5% CO_2_ (19,20). Cells were then incubated with anti-CD14 mAbs conjugated with PerCP/Cy5.5 (0.3 μL), anti-CD1a-FITC (1 μL), anti-CD83-PE (1 μL), and anti-CD11c-APC (1 μL) for 30 min (Biolegend). A Fluorescence Minus One (FMO) control was performed. DCs phenotyping protocol was carried out to confirm cell differentiation, and cells were analyzed in the FACS CaliburTM flow cytometer acquiring a total of 50,000 events. DCs were accordingly generated, presenting the typical cell markers CD11c^high^, CD1a^high^, CD83^low^, and CD14^low^ (21).

DCs were incubated with propolis alone or in combination with EtxB or LPS diluted in RPMI 1640 supplemented with 10% FBS (complete medium) for 48 h in the following protocols.

### Dendritic cell viability

Cell viability was performed using the MTT [3-(4,5-dimethyl-thiazol-2-yl)-2,5 diphenyltetrazolium bromide] (Sigma-Aldrich) colorimetric assay.

DCs were incubated with the stimulants in a final volume of 100 μL. The supernatants were removed and 100 μL of MTT (1 mg/mL) in complete RPMI was added to the culture cells. After 3 h, MTT was removed and 100 μL of DMSO was added. The absorbance was read at 540 nm and the percentage of cell viability was calculated using the formula: [(absorbance test / absorbance control) × 100].

### T lymphocyte proliferation

**Figure 1:**
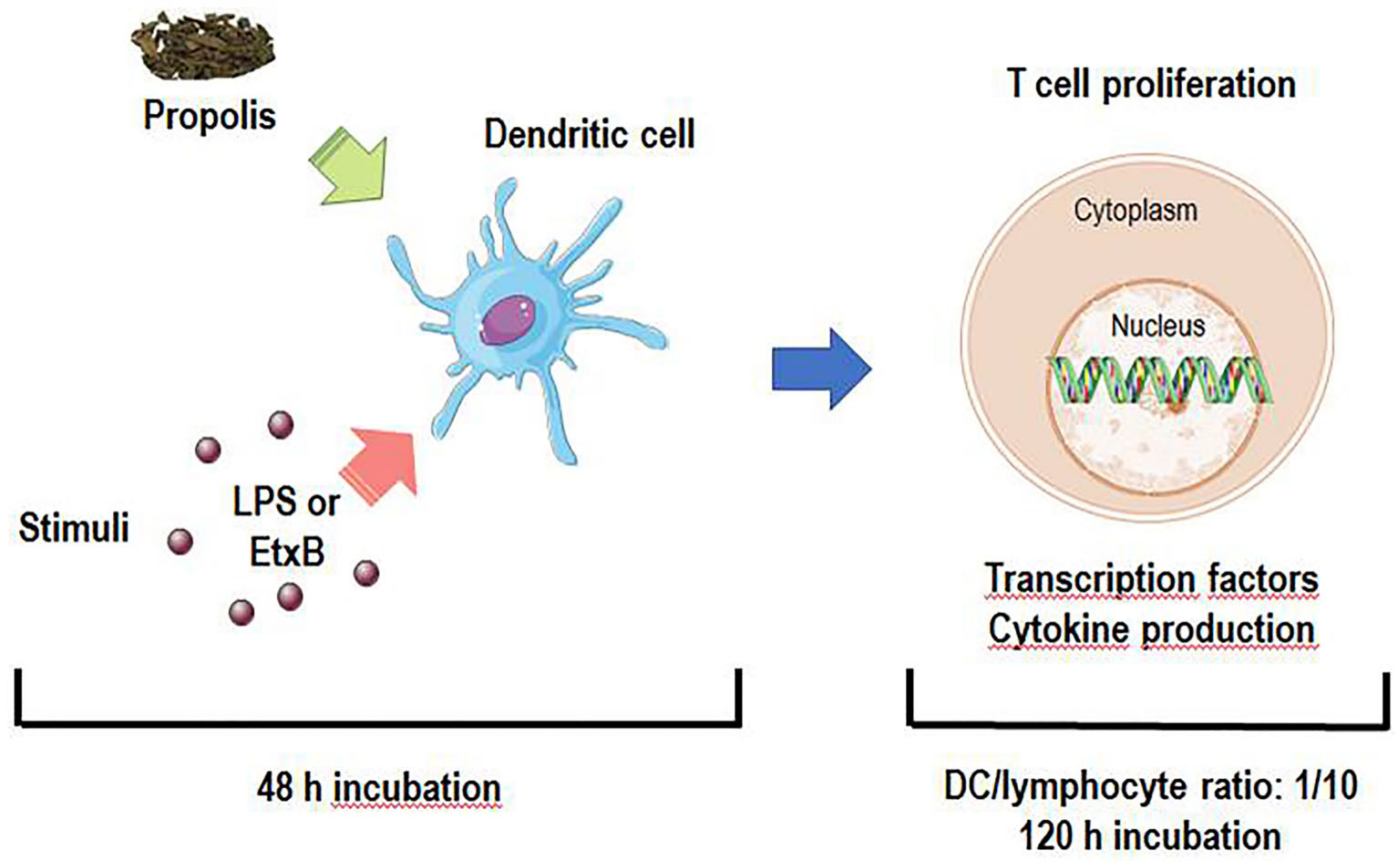
Experimental design. Dendritic cells (DC, 1×10^6^ cells/mL) were incubated with propolis, lipopolysaccharide (LPS), enterotoxin B subunit (EtxB), or their combination. After 48 h, supernatants were discarded, cell cultures were washed and then incubated with autologous CD4^+^ T lymphocytes (DCs/lymphocyte ratio = 1/10) for 120 h to assess lymphocyte proliferation, transcription factors expression (*GATA-3* and *RORc*), and cytokine production (interleukin (IL)-4 and IL-17A).

To assess lymphocyte proliferation, isolated CD4^+^ T cells were labeled with carboxy-fluorescein succinimidyl ester (CFSE) (Cell-Trace CFSE Proliferation Kit, Molecular Probes, Invitrogen, USA). For co-culture assays, DCs incubated with EtxB or LPS with or without propolis for 48 h were washed and incubated with CFSE-labeled autologous CD4^+^ T lymphocytes (DCs/lymphocyte ratio=1/10) for 120 h (Figure 1). Phytohemagglutinin (PHA, 2.5 μg/mL) was used as a positive control for cell proliferation, and cells without any marking (autofluorescence) were used as a negative control, in addition to FMO control under the same conditions. After incubation, lymphoproliferation was evaluated in the FACS CaliburTM flow cytometer, acquiring a total of 50,000 events.

### *GATA-3* and *RORc* expression

CD4^+^ T lymphocytes were evaluated by expressing the genes encoding *GATA-3* and *RORc* transcription factors. After treating DCs with propolis, EtxB, or LPS for 48 h, cells were incubated with lymphocytes for 120 h. Then, total RNA was extracted using the RNeasy Mini Kit (Qiagen, Netherlands). The isolated RNA was treated with RQ1 RNase-Free DNase (Promega, USA), and cDNA synthesis was performed with the ProtoScript II Reverse Transcriptase kit (BioLabs, USA), according to the manufacturer’s instructions. For RT-qPCR, the GoTaq-qPCR Master Mix (Promega) was used and the primer sequences are listed in Table 1. Each reaction was performed in triplicate under the following conditions: 50°C for 2 min, 95°C for 10 min for initial denaturation, 40 cycles at 95°C for 15 s, and 60°C for 6 s, followed by the melting curve. The expression values of the analyzed transcripts were normalized with glyceraldehyde-3-phosphate dehydrogenase (*GAPDH*). The calculation of the differential expression of the selected genes was performed using a standard curve (22). For analysis of relative gene expression, all samples were standardized in relation to an RNA sample with a relative value of 100.

**Table 1:** Gene and primer sequences.

Gene ID	Primer 5'-3' sequence	GeneBank
*GATA-3*	Forward primer: (174) CTCTTCGCTACCCAGGTGAC (193)	NM_001002295.1
	Reverse primer: (269) ACGACTCTGCAATTCTGCGA (250)	
*RORc*	Forward primer: (363) CATGTCCCGAGATGCTGTCA (382)	NM_005060.3
	Reverse primer: (473) GGTTCCTGTTGCTGCTGTTG (454)	
*GAPDH*	Forward primer: (684) CGTGGAAGGACTCATGACCA (703)	NM_002046.4
	Reverse primer: (801) GGCAGGGATGATGTTCTGGA (782)	

### Assessment of intracytoplasmic cytokines

Six hours before the end of co-culture incubation, brefeldin A (BFA; Biolegend) was added to prevent the release of cytokines from the cytoplasm. The cells were labeled with anti-CD4 conjugated to PerCP/Cy5.5 (OKT4 clone; Biolegend), which allowed the selection of the gate of only the CD4^+^ lymphocytes. The cells were then incubated with a solution of Fix & Perm Cell Fixation and Permeabilization kit (Nordic MUbio, The Netherlands) in the presence of anti-IL-4 conjugated with PE (clone MP4-25D2; Biolegend) and PE-conjugated anti-IL-17A (clone BL168; Biolegend). After incubation, the cells were analyzed by flow cytometry and, for each test, an isotypic control with the respective test fluorochromes (PerCP/Cy5.5 and PE; Biolegend), an autofluorescent control, and FMO controls were included. Analyses were performed using the FACS CaliburTM flow cytometer and the FlowJo software v. X.0.7 (https://www.flowjo.com). A total of 50,000 acquisition events were standardized per sample, and the population of interest was optimized by establishing a gate based on size (FSC) and granularity (SSC) parameters. The results are reported as the percentage of CD4 positive cells expressing IL-4 or IL-17A.

### Statistical analysis

The Shapiro-Wilk test was used as a normality test. All data were analyzed from 5 individuals in triplicate and are reported as means±SD. Data analysis was performed using ANOVA with the Graph Pad Prism 5 software (USA). The data were further analyzed with Dunnett's test and differences were considered statistically significant at P<0.05.

## Results

### Cell viability

**Figure 2:**
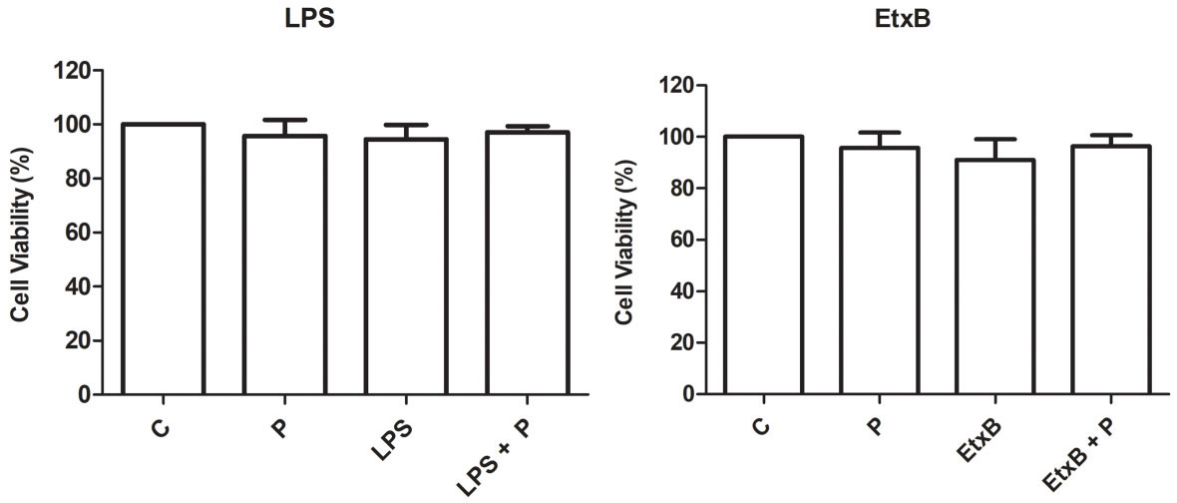
Viability (%) of dendritic cells (1×10^6^ cells/mL) after 48 h incubation with RPMI 1640 (control, C), propolis (P, 5 μg/mL), lipopolysaccharide (LPS, 1 µg/mL), enterotoxin B subunit (EtxB, 2 µg/mL), or their combination. Data are reported as means±SD of 5 subjects. P>0.05 (ANOVA).

Before incubating the co-cultures, a possible cytotoxic effect of propolis and the stimuli on DCs was analyzed by MTT assay. The treatments did not exhibit any cytotoxic effect on these cells (Figure 2). The solvents used for EtxB (ultrapure water) and propolis (70% ethanol) did not affect DCs viability as well (data not shown).

### T lymphocyte proliferation

**Figure 3:**
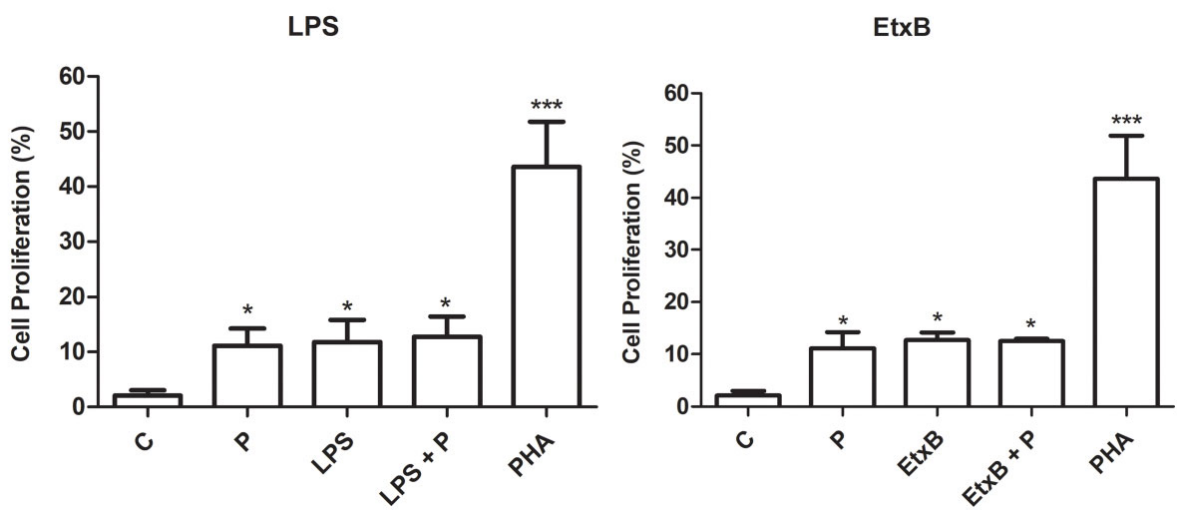
Percentage (%) of lymphocyte (1×10^6^ cells/mL) proliferation after 120 h of co-culture with autologous dendritic cells treated with RPMI 1640 (control, C), phytohemagglutinin (PHA, 2.5 μg/mL), propolis (P, 5 μg/mL), lipopolysaccharide (LPS) (1 µg/mL), enterotoxin B subunit (EtxB, 2 µg/mL), or their combination for 48 h. Data are reported as means±SD (n=5). *P<0.05, ***P<0.001 compared to control (ANOVA).

All treatments increased proliferation after lymphocyte co-culture with DCs treated with propolis as well as with LPS or EtxB, alone or in combination (P<0.05), compared to co-cultures with untreated DCs (Figure 3).

### *GATA-3* and *RORc* gene expression

**Figure 4:**
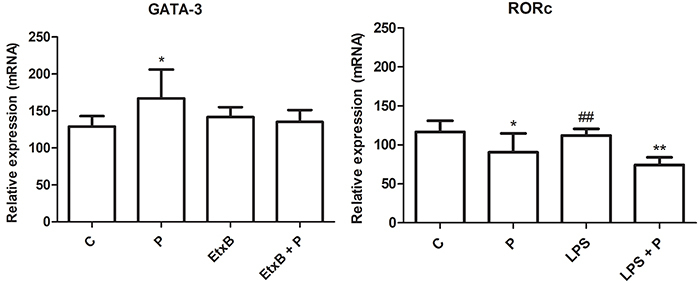
*GATA-3* and *RORc* relative expression by lymphocytes after 120 h of co-culture with autologous dendritic cells treated with RPMI 1640 (control, C), propolis (P, 5 μg/mL), enterotoxin B subunit (EtxB, 2 µg/mL), lipopolysaccharide (LPS, 1 µg/mL), or their combination. Data are reported as means±SD (n=5). *P<0.05, **P<0.01 compared to control; ^##^P<0.01 compared to LPS+P (ANOVA).

Propolis stimulated *GATA-3* expression (P<0.05), while the B subunit of the *E. coli* thermolabile enterotoxin (EtxB), in the presence or absence of propolis, did not alter its expression (Figure 4).

Propolis inhibited *RORc* expression (P<0.05). LPS did not affect *RORc* expression, but LPS in combination with propolis inhibited it (P<0.01).

### Cytokine production

**Figure 5:**
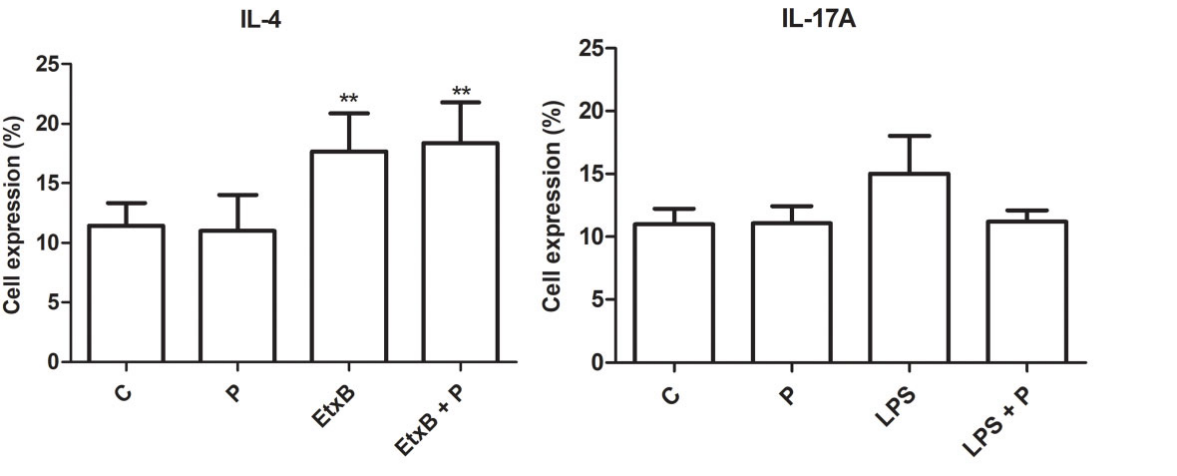
Percentage (%) of lymphocytes expressing interleukin (IL)-4 and IL-17A after 120 h of co-culture with autologous dendritic cells treated with RPMI 1640 (control, C), propolis (P, 5 μg/mL), enterotoxin B subunit (EtxB, 2 µg/mL), lipopolysaccharide (LPS, 1 µg/mL), or their combination. Data are reported as means±SD (n=5). **P<0.01 compared to control (ANOVA).

Propolis alone did not affect IL-4 production. EtxB alone or in combination with propolis induced IL-4 production (P<0.01). LPS alone did not increase IL-17A and its combination with propolis maintained baseline levels (Figure 5).

## Discussion

Upon recognition of antigens, DCs are able to process and present epitopes to further interact with naive T lymphocytes and trigger apoptosis, anergy, tolerance, or activation of T helper profiles that determine the expansion, regulation, or suppression of the immune response (23,24). Current immunological experiments have been conducted involving different conceptual perspectives and experimental designs and, in this work, we investigated the mechanisms modulated by propolis in the phenotype and functions of T CD4^+^ cells activated by EtxB or LPS-stimulated DCs, because CD4^+^ T cells are the major orchestrators of the adaptive immune response (6).

It is evident that DCs play a key role in the induction of an inflammatory response as well as in the activation of adaptive immunity. Understanding the general and specific functions of DCs is useful to characterize the pattern of inflammation triggered by the adaptive immune response and represents a promising strategy for the development of immunotherapies for inflammatory conditions. To investigate the dynamics of DCs in T cell activation, we first investigated lymphocyte proliferation. A higher proliferation was seen after DCs were treated with propolis, LPS, or EtxB compared to untreated DCs. Transcription factor expression and cytokine production were next evaluated.

GATA-3 transcription factor promotes the differentiation of Th2 cells and stimulates the expression of Th2 cytokines, such as IL-4, IL-5, IL-13, and others. Propolis alone significantly induced the expression of *GATA-3* without affecting IL-4 production. EtxB, in the presence or absence of propolis, did not alter *GATA-3* expression but induced IL-4 production. This toxin is a potent immunomodulator that activates the Th2 profile and the polyclonal activation of B cells and modulates the activity of Treg cells (25,26).

While propolis alone induced the expression of *GATA-3*, it decreased the expression of *RORc*, which is related to the Th17 profile. Propolis did not affect IL-17 production. Interestingly, LPS and LPS combined with propolis reduced *RORc* expression. LPS alone increased the percentage of cells expressing IL-17A and its combination with propolis maintained the baseline levels of these cytokines. One may speculate that the kinetics of gene expression and cytokine production took place at different times, so that no correlation was seen between such parameters. LPS has a positive effect on the differentiation of the Th17 profile *in vitro* due to its ability to induce the production of inflammatory cytokines (11). McAleer and coworkers (27) observed equivalent proportions of Th17 and Th1 cells in the intestinal lamina propria of mice after intraperitoneal immunization using LPS as an adjuvant.

Our data showed that propolis may enable the differentiation of the Th2 profile and inhibit the Th17 profile. In fact, propolis has attracted the interest of researchers investigating its effects on the immune system, especially for increasing antibody production in combination with vaccines and for its therapeutic use in inflammatory conditions (17,28). This is in agreement with data obtained with experimental animals (rats, mice, fish, and birds) treated with propolis and immunized with different vaccines/antigens, showing higher titers of specific antibodies compared to animals that received only the antigens (29-35).

Th17 cells play an important role in adaptive immunity and protect the host against several pathogens. Microbial antigens may induce the expression of IL-23, which stimulates the differentiation and activation of the Th17 profile by the main regulator RORγt/RORc and favors chronic inflammatory diseases, making them an important target for the treatment of inflammatory disorders (36,37). The effect of propolis was investigated in animal models, and a decreased IL-17 production was observed in murine splenocytes stimulated *in vitro* with PMA in the presence of propolis in a dose-dependent manner. *In vivo*, DBA/1J mice with collagen-induced arthritis treated with propolis showed better scores than animals treated only with standard diet, as well as a decrease in the number of IL-17, IFN-γ, and IL-4 secretory cells, suggesting that propolis suppresses the differentiation of Th17 cells (34). Our findings are in agreement with those of Santiago et al. (38), who reported that propolis affected Th1 responses and led naive T cells to a regulatory profile, reinforcing the potential use of propolis for the treatment of inflammatory conditions.

The progress made through studies to develop new drugs and the interest in the biological properties and therapeutic applications of propolis are expressive (17). Although it is still a challenge to achieve universal standardization, research on the chemical composition and pharmacological properties of propolis has revealed its positive effects and possible targets for therapeutic use. Evidence has pointed out the potential of propolis and its constituents for the development of new anti-inflammatory drugs, involving mechanisms related to inhibition of cytokines and chemokines, intracellular signaling pathways, adhesion, and cell migration (16,39,40). Our data contributed to understanding the underlying mechanisms in DC activation in favor of Th2 *vs* Th17 differentiation, expanding the potential immunomodulatory role of propolis in the functions of these cells, and supporting the development of new drugs to treat inflammatory diseases.

Altogether, propolis can affect the crosstalk between DCs and CD4^+^ T cells, favoring a Th2 profile that may culminate in the humoral immunity against extracellular antigens and inhibiting the Th17 profile. These results open perspectives for the study of certain biological events involving propolis in the treatment of inflammatory/autoimmune diseases.
